# Increased prokaryotic diversity in the Red Sea deep scattering layer

**DOI:** 10.1186/s40793-023-00542-5

**Published:** 2023-12-14

**Authors:** Tamara Megan Huete-Stauffer, Ramiro Logares, Mohd Ikram Ansari, Anders Røstad, Maria Lluch Calleja, Xosé Anxelu G. Morán

**Affiliations:** 1 Red Sea Research Center, Blg 2, Level 2, Office 2217-WS05, BESE, King Abdullah University of Science and Technology (KAUST), Thuwal, 23955-6900 Kingdom of Saudi Arabia; 2 Institute of Marine Sciences (ICM), CSIC, Barcelona, Spain; 3 Department of Biosciences, Integral University, Lucknow, Uttar Pradesh India; 4 Marine Ecology and Systematics, Biology Department, University of the Balearic Islands (UIB), Palma, Spain; 5 Centro Oceanográfico de Gijón/Xixón (IEO), CSIC, Gijón, Spain

**Keywords:** Marine microbial ecology, Mesopelagic, Deep scattering layer, Diel vertical migration, Red Sea microbiome, rRNA diversity

## Abstract

**Background:**

The diel vertical migration (DVM) of fish provides an active transport of labile dissolved organic matter (DOM) to the deep ocean, fueling the metabolism of heterotrophic bacteria and archaea. We studied the impact of DVM on the mesopelagic prokaryotic diversity of the Red Sea focusing on the mesopelagic deep scattering layer (DSL) between 450–600 m.

**Results:**

Despite the general consensus of homogeneous conditions in the mesopelagic layer, we observed variability in physico-chemical variables (oxygen, inorganic nutrients, DOC) in the depth profiles. We also identified distinct seasonal indicator prokaryotes inhabiting the DSL, representing between 2% (in spring) to over 10% (in winter) of total 16S rRNA gene sequences. The dominant indicator groups were *Alteromonadales* in winter, *Vibrionales* in spring and *Microtrichales* in summer. Using multidimensional scaling analysis, the DSL samples showed divergence from the surrounding mesopelagic layers and were distributed according to depth (47% of variance explained). We identified the sources of diversity that contribute to the DSL by analyzing the detailed profiles of spring, where 3 depths were sampled in the mesopelagic. On average, 7% was related to the epipelagic, 34% was common among the other mesopelagic waters and 38% was attributable to the DSL, with 21% of species being unique to this layer.

**Conclusions:**

We conclude that the mesopelagic physico-chemical properties shape a rather uniform prokaryotic community, but that the 200 m deep DSL contributes uniquely and in a high proportion to the diversity of the Red Sea mesopelagic.

**Supplementary Information:**

The online version contains supplementary material available at 10.1186/s40793-023-00542-5.

## Background

The diel vertical migrations of organisms (fish and zooplankton) between the surface and the mesopelagic layers provide a mechanism for the enrichment and transport of labile dissolved and particulate organic matter (DOM and POM) to the deep ocean [1–4]. The migrating organisms concentrate in deep waters during the day and swim to the more productive surface waters to feed during the night. At depth, they assemble in layers that reflect sound from echosounders thus receiving the name of deep scattering layers (DSL). This migrative behavior is widespread across all oceanic basins [4–7], but the intensity of the migration, the depth of the scattering layers and the organisms involved vary widely.

The oligotrophic and tropical Red Sea represents one of the most extreme cases of DVM observed [8]. Over 95% of the entire populations of fish at the DSL participate in the daily migration [8–10], compared to 20–90% in other oceanic areas [5], representing a tenfold change in biomass from day to night [9].

There are two DSLs in the Red Sea that can be observed between 400 and 800 m [8, 9], with varying depths and widths depending on the time of day and light intensity. The first DSL is composed almost exclusively of the species *Vinciguerria* sp. (a type of lightfish) and the second of *Benthosema pterotum* [11], a small lanternfish 2–7 cm long [9, 12]. The drastic migratory behavior of these fish departs from observations in other areas due to 2 inherent characteristics of the Red Sea: the low abundances of zooplankton and the high temperatures present in the mesopelagic waters.

The DSLs generally have a mixed composition of fish and zooplankton, especially in productive basins [9, 13, 14]. In these cases, a fraction of the fish population can stay at depth and feed on the deep zooplankton [5, 13] but the biomass of zooplankton in the deep Red Sea is too low to support the fish populations [9, 15]. On the other hand, reduced and shallow water exchanges through the Gulf of Aden and high irradiances generate water temperatures of up to 34 °C in surface waters of the Red Sea [16]. As a consequence of the permanent stratification and limited cooling processes, the deep water masses exhibit temperature values of almost 22 °C below 200 m [17, 18], extremely high compared to other mesopelagic basins. The high temperatures observed in the deep Red Sea accelerate the digestive metabolism of the mesopelagic fish, which combined with the low amount of zooplankton in mesopelagic waters likely explain the drastic migrations observed [8, 9].

The deepest DSL (450–600 m) represents a recently documented hotspot for microbial activity and diversity in the deep ocean [1, 19]. The organic matter consumed during the night by the migrating organisms is transported downwards every sunrise to the DSL. A fraction of this organic matter is metabolized and released during the day as fecal pellets and dissolved substrates in the DSL, generating potentially large inputs of particulate and dissolved organic matter. Fish-mediated carbon export has been reported to transport up to 40% of surface primary production [20] or even higher [4]. Particularly in the Red Sea, a recent modeling approach concludes that DVM was responsible for 32% of the total carbon flux to deep layers and significantly enhanced carbon sequestration by 36% [21]. Excretion products include ammonium [22], amino acids and fatty acids [23]. In turn, excretion products can trigger both heterotrophic and autotrophic prokaryotic metabolism which are key to the cycling of nutrients and carbon in the ocean, especially in deep waters, where energy sources are scarce [24, 25] and remineralization processes mediated by bacteria and archaea are essential to ensure the survival and growth of higher trophic levels. Also, the additional inputs of DOM may promote prokaryotic respiration that can alter the carbon budgets and affect global estimates of carbon export [26].

The prokaryotic diversity of the DSL remains virtually unexplored despite its potential global importance for carbon and nutrient cycling and its role as a microbial hotspot. We are aware of very few studies exploring the microbial community at this layer and they have only been published in the past couple of years [1, 27]. In our research, we gathered samples from three Red Sea cruises to conduct a comprehensive analysis of the DSL diversity and its environmental context. Our goal was to compare this data with the broader water column, encompassing both epipelagic and mesopelagic zones around the DSL. Additionally, we aimed to investigate the potential influence of fish diel migrations on prokaryotic diversity in the mesopelagic realm.

## Methods

### Sampling

**Fig. 1:**
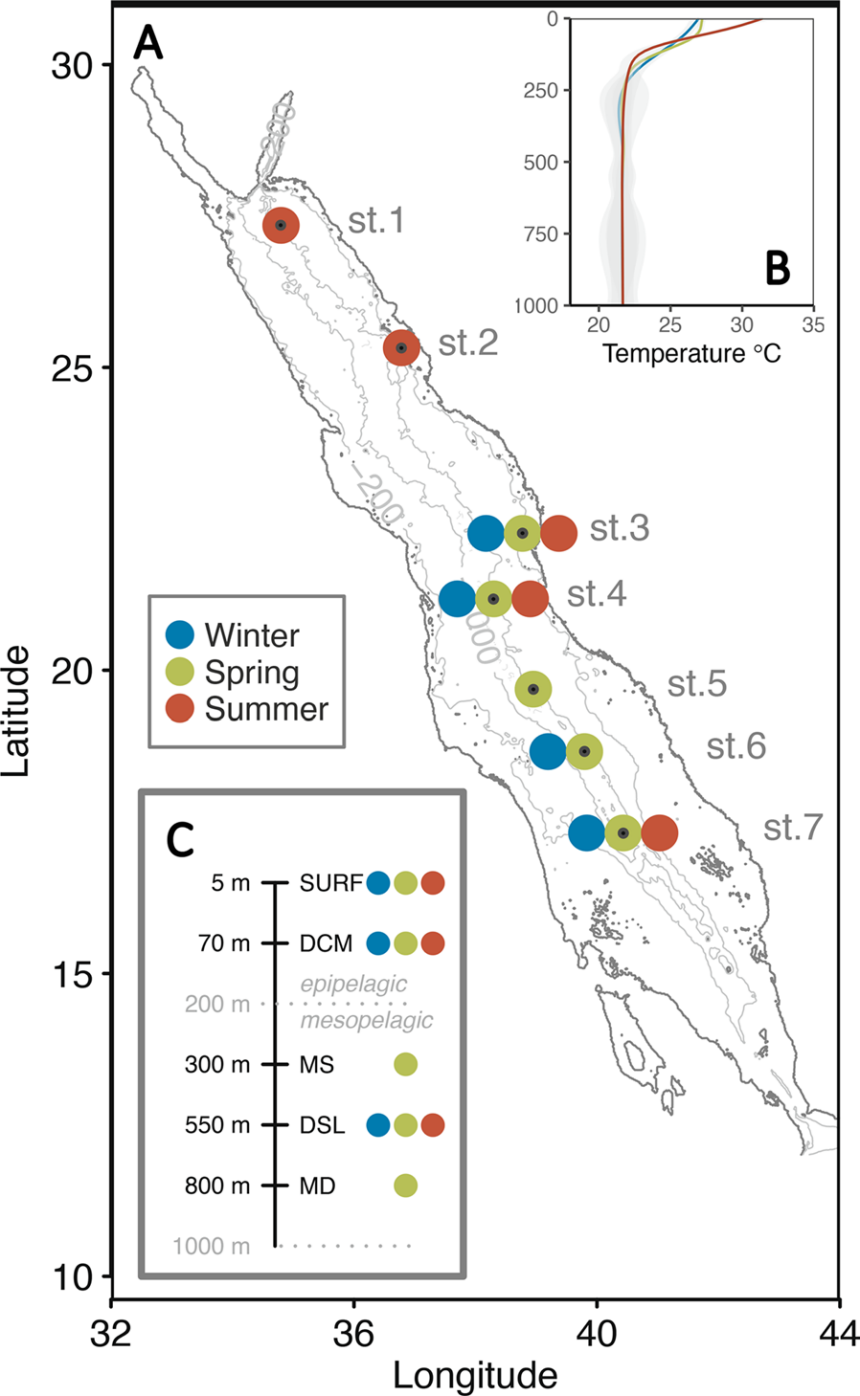
Sampling strategy. **A** Location of the stations sampled along the Red Sea colored according to the seasons when samples were collected (winter, spring and summer). The black dot indicates the exact coordinates of collection. **B** Average temperature profile by season **C** Layers sampled in each season, indicating the average depth of collection (SURF, DCM, MS, DSL, MD)

Samples were collected along the central axis of the Red Sea (Fig. 1) during 3 consecutive cruises: winter 2017 (31 Jan–7 Feb), on board of *R/V Thuwal*, summer 2017 and spring 2018 (2–16 Aug and 16–21 Mar, respectively) on board *R/V of Al Azizi*. At each designated station, a SeaBird 9 or Idronaut CTD mounted on a 12 bottle (10 or 25 L) rosette was deployed, measuring continuously temperature, salinity, fluorescence (ECO-AFL/FL, Wet Labs, calibrated with chlorophyll *a*) and dissolved oxygen. Salinity and oxygen profiles from 4 stations of the spring cruise were partially lost due to CTD malfunction and were reconstructed at 1 m bins using Red Sea casts available from the previous years at the same stations or at similar coordinates (in total 36 profiles were used). This was possible since the salinity profiles are quite constant at specific latitudes. At all stations, 13 depths (from 5 to 1000 m) were sampled for inorganic nutrients, DOC, fluorescent DOM, and prokaryotic abundance and cell size. Water for prokaryotic DNA analysis was collected at the surface (SURF: 5 m), deep chlorophyll maximum (DCM: 65–98 m) and deep scattering layer (DSL: 490–600 m). Additionally, during the spring cruise, samples were also collected above the DSL in the shallow mesopelagic (MS, 250–300 m) and below the DSL in the deep mesopelagic (MD, 750–900 m) (Fig. 1C and Additional file 1: Fig. S1). All contextual variables were measured at the depths where DNA samples were collected.

### Acoustics

The position of the DSL at each station was established using 38 kHz echosounders. On the *R/V Thuwal* a hull-mounted Simrad EK60 was used. On the *R/V Al Azizi* a portable Simrad WBAT was deployed at 5 m depth at the stern of the vessel. Both systems had a 38 kHz Simrad transducer with a 7-degree opening angle. We targeted the most intense DSL, normally the deepest one (typically composed of *Benthosema pterotum*), but sometimes sampled the layer above (Additional file 1: Fig. S1). Sampling was performed during the day, at around noon (12:00 PM) to ensure the fish were concentrated at the DSL.

### DOC

Samples above 200 m were prefiltered through a pre-combusted glass fiber filter (Whatman GF/F, 0.7 µm nominal pore size), while samples below 200 m were collected directly from the Niskin’s nozzle. Water was collected in opaque acid washed and pre-combusted 40 mL glass vials, acidified with orthophosphoric acid to pH 1–2 and stored at 4 °C until processing in a Shimadzu TOC-L analyzer. Briefly, samples were analyzed through high temperature catalytic oxidation (HTCO) and measured against consensus deep water standards (42–45 µmol C L^−1^ and 31–33 µmol N L^−1^) and low carbon water (1–2 µmol C L^−1^), obtained from D. A. Hansell (Univ. of Miami).

### Biological index of DOM

Samples for fluorescence of DOM were collected in 125 mL dark polycarbonate bottles. Fresh samples were analyzed in a HORIBA Jobin Yvon AquaLog spectrofluorometer with a 1 cm path length quartz cuvette. The UV–Vis fluorescence was recorded in excitation-emission matrices (EEMs) that covered the range 240–600 nm of excitation and 250–600 nm of emission wavelengths, both at 3 nm increments and integration times of 8 s. Calibration, correction and analysis of EEMs followed the same steps as in [25]. The biological index (BIX) was obtained as the ratio of emission at 380 and 430 nm at 310 nm of excitation wavelength [28] and is an indicator of recent autotrophic productivity, with values close or higher to 1 revealing recently produced DOM of autochthonous origin [29].

### Inorganic nutrients

**Fig. 2:**
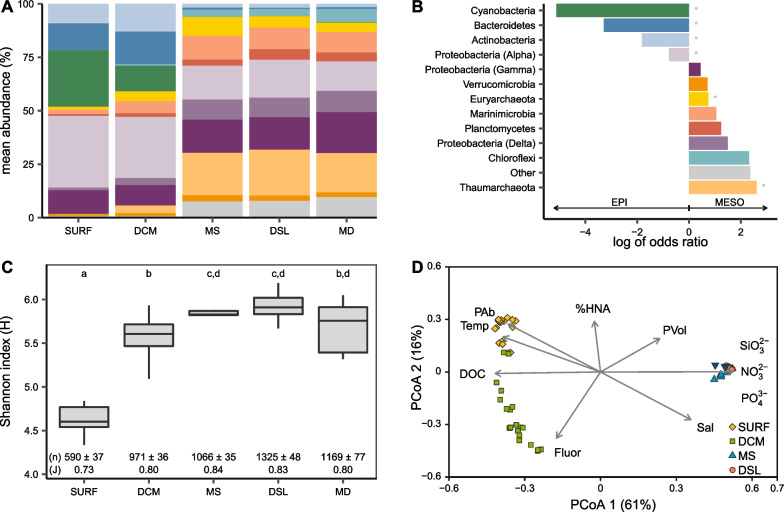
Red Sea prokaryotic diversity. **A** Mean sequence relative abundances of the top most abundant phyla (over 1%) in the different layers (SURF, DCM, MS, DSL, MD). Proteobacteria has been further divided into the most abundant classes. **B** Log of odds ratio of the top most abundant phyla plus Proteobacteria classes. A positive value indicates that a phylum has higher probability of being observed in the mesopelagic (mean of MS, DSL and MD), while a negative value indicates a higher probability of appearing in the epipelagic zone (mean of SURF and DCM). Asterisks represent significant odds ratios (p < 0.05) calculated with Fisher’s exact test. **C** Boxplot representing the alpha diversity index of Shannon. Annotations under each box show richness as number of species (n) and Pilou’s evenness (J). Letters at the top of the figure indicate the different groups according to ANOVA tests on all 3 indexes (H, n, J). **D** Principal Coordinates Analysis of Bray–Curtis distances of all the 67 samples considered in the study, color-coded by layer. Scaled (zero-centered) environmental and biological variables that have a significant effect on the multidimensional distribution are shown as arrows. The arrow tip indicates the direction where the effect is maximum and the length indicates the magnitude of the effect (Fluor = CTD chlorophyll *a* fluorescence, DOC = dissolved organic carbon, Temp = temperature, PAb = prokaryotic abundance cells mL^−1^, %HNA = percentage of HNA cells, PVol = prokaryotic volume µm^3^, NO_3_^2−^ = nitrate µmol L^−1^, PO_4_^3−^  = phosphate µmol L^−1^, SiO_3_^2−^  = silicate µmol L^−1^, Sal = salinity)

Duplicate samples were collected in clean 15 mL Falcon tubes and frozen at −20 °C until analysis. Nitrate (NO_3_^−^), nitrite (NO_2_^−^), phosphate (PO_4_^3−^) and silicate (SiO_2_) were analyzed in a SEAL AA3 segmented flow analyzer (Seal Analytical) using standard methods [30]. All standards were prepared with a nutrient-free artificial seawater matrix in acid-washed glassware. NO_3_^2−^ was highly correlated to SiO_3_^2−^ and PO_4_^3−^ (r = 0.978 and r = 0.985, respectively, p < 0.001, n = 67) and has been used in subsequent analysis as a representative of inorganic nutrients to avoid redundancy of environmental parameters (PCoA in Fig. 2D).

### Prokaryotic abundance and size

1.8 mL of unfiltered seawater were collected and fixed with a final concentration of 1% paraformaldehyde and 0.05% glutaraldehyde, flash frozen in liquid nitrogen and stored at −80 °C until analysis. An aliquot of 400 µL was thawed and stained with SYBR Green I at 100× final concentration. After adding 1 µm fluorescent beads as a reference of fluorescence and size, samples were analyzed on a FACSCanto flow cytometer at a low flow rate (18–33 µL min^−1^, measured empirically every day) for 2 min or until 10,000 events were achieved. Total prokaryotes and high and low nucleic acid cells (HNA and LNA, respectively), were identified simultaneously on plots of green fluorescence (SYBR Green I emission wavelength) against side scatter (a proxy of size) and green fluorescence against red fluorescence (chlorophyll *a* emission wavelength), to separate cyanobacteria from heterotrophic prokaryotes in the upper layers. Cell counts were converted to cells mL^−1^ and side scatter was converted to diameter by applying the empirical calibration of Calvo-Díaz and Morán (2006).

### DNA collection, extraction and sequencing

5 L (SURF and DCM) or 9 L (MS, DSL, MD) of seawater were collected in clean acid-washed polycarbonate carboys and filtered through 0.2 µm Sterivex filters. Samples were flash-frozen in liquid N_2_, preserved at −50 °C during the cruise and stored at −80 °C until extraction.

DNA extraction on 67 samples was preformed using the PowerSoil DNAeasy kit from MoBio/Qiagen after carefully releasing the Sterivex filters from their capsule, keeping a sterile environment. Diversity was estimated from the amplification of the V4-V5 region of the 16S rRNA gene using the primers 515F-Y and 926R [31] with Illumina Nextera attached adapters. The first amplification PCR was performed in triplicate 10 µL reaction mixtures containing 1 ng of DNA, 0.2 µM of each of the barcoded primers, 0.16 µM of AccuStart Taq DNA polymerase and 5 µM of 1X FailSafe PCR premix (Epicenter, Illumina). Cycling conditions included a 3 min heating step at 95 °C followed by 25 cycles of 95 °C for 45 s, 50 °C for 45 s, 68 °C for 90 s, and a final extension of 68 °C for 5 min [31]. The triplicate PCR products were pooled for subsequent downstream analysis, following the MiSeq Illumina protocol. Briefly, the PCR product was purified using magnetic Ampure XP beads (Beckman Coulter, Brea, CA, USA), then a library was created attaching to each sample a unique combination of Nextera indexes by PCR, also purified with magnetic beads. All samples were pooled at equimolar concentrations and the pooled 16S band was purified from an agarose gel with Wizard gel + PCR Clean-up (Promega) to remove any remaining contaminants and PCR artifacts. The clean pool was quantified using KAPA SYBR FAST Universal qPCR kit with Illumina Primer Premix (Kapa Biosystems Ltd., London, UK) and the average DNA strand size was assessed using a Bioanalyzer (Agilent Technologies, Santa Clara, USA). 6 pM of the gene amplicon libraries were sequenced on one lane on Illumina MiSeq platform with 25% PhiX control at the Bioscience Core Lab at KAUST. The libraries were sequenced using 2 × 300 bp overlapping paired-end reads using Illumina MiSeq V3 kit.

### Sequence processing

The raw sequences are available at https://www.ebi.ac.uk/ena/browser/view/PRJEB49545 as 67 paired fastq sequences with consecutive accession numbers: ERX7411972–ERX7412038. The Nextera indexes and primers were trimmed from the raw sequences using cutadapt 2.3 [32]. The clean sequences were processed with the DADA2 pipeline [33, 34] trimming the forward read at 220 bp and the reverse read at 200 bp, that rendered a total of 12,502 unique amplicon sequence variants (ASVs). ASVs are obtained by denoising instead of by clustering methods (that give traditional OTUs) and seem to represent real sequences more accurately [35]. The taxonomic affiliation from domain to genus level was assigned based on the RDP Classifier [36] against the reference database SILVA132 with at least 80% bootstrap confidence while species were only assigned when the sequence resulted in an exact match to reference strains. ASVs not assigned as Archaea or Bacteria (e.g., Chloroplasts, Mitochondria, other taxa) as well as singletons were removed and samples were normalized to the minimum number of reads (82,130). The final number of ASVs was 8889. All analyses were performed with the normalized table. In this text we use interchangeably the term *ASV* and *species* for simplicity, but it is possible that some ASVs can belong to the same species with hypervariable V4/V5 regions or vice versa, the same ASV could belong to different species with low variability in the V4/V5 region [37]

### Statistical analyses and programming resources

All analyses were performed in R 3.6.1 [38] within RStudio 1.1.447 (RStudio, Inc.) using the packages: *tidyverse* [39] for overall scripting; *dada2* [33] for sequence processing; *vegan* [40] for multidimensional analyses (PCoA, CCA), non-parametric analysis of variance (PERMANOVA), zero centering of continuous variables, dissimilarity matrices and diversity indexes; *pairwiseAdonis* [41] for adjusted p-values of PERMANOVA pairwise comparisons; *labdsv* [42] for calculations of indicator species; *flowCore* [43, 44] for flow cytometry data analysis; *corrplot* [45] for correlation matrices; *cowplot* [46], *gplots* [47] and *ggplot2* [48] for visualization and arrangement of figures; *marmap* [49] for downloading and plotting NOAA terrestrial and bathypelagic data. Unless otherwise mentioned, statistical significance is considered when p < 0.05.

## Results

### Oceanographic conditions

Temperature-salinity diagrams of the CTD casts were performed to identify the water masses present during the study (Additional file 1: Fig. S2). The water in the epipelagic (i.e., above isopycnal 27.5 kg m^−3^) was more variable in its thermohaline properties, showed clear seasonal variability and belonged to the Red Sea Surface Water. The mesopelagic (below isopycnal 27.5 kg m^−3^) belonged to the Red Sea Deep Water (RSDW), a very well-characterized and homogeneous water mass from 200–250 m to the bottom with temperatures of 21.7 °C, salinity of 40.6 and density of between 27.5 and 28.4 kg m^−3^ [18, 50]. During our study, the isopycnal 28.4 kg m^−3^ was located at a mean depth of 248 m. Below that depth, density variations were negligible regardless of seasons. During our sampling period (over a time span of 1.5 years), no intrusions of water masses different from the RSDW were evident (Additional file 1: Fig. S2).

The depth profiles of relevant variables are shown in Additional file 1: Fig. S3, and showed that some physico-chemical properties (DOC, oxygen and especially nitrate and phosphate concentrations) varied with season, especially in winter.

### Depth profiles of 16S rRNA gene

The samples were collected along the latitudinal axis of the Red Sea in 3 cruises (Fig. 1) as part of a survey targeting the strong, opposite gradients in temperature and salinity found from North to South [51, 52]. No significant differences were observed between the prokaryotic diversity profiles and the different station locations (pairwise PERMANOVA p > 0.05 performed on the ASV matrix including station, season and layer as factors). Thus, latitudinal effects were not further considered in this study. We proceeded to compare the profiles of the different stations independently from their actual location.

The overall prokaryotic diversity in our Red Sea samples is shown in Fig. 2. In total, 40 different phyla were identified. The relative abundance of the 10 most abundant phyla (Fig. 2A) showed clear differences in the dominant clades between the epipelagic (SURF and DCM) and the mesopelagic (MS, DSL and MD) samples. A detailed view of the top phyla at each individual station is shown on Additional file 1: Fig. S4 and a complete list of observed phyla is shown in Additional file 1: Fig. S5. We used the log of odds ratio to compare and quantify the proportions of each of the dominant clades and retrieve a probability of presence in the mesopelagic (positive values) or in the epipelagic (negative values). *Actinobacteria*, *Bacteroidetes* and *Cyanobacteria* represented on average 45% of the total abundance in the epipelagic (11%, 15% and 19%, respectively) and showed very low probabilities of being found in the mesopelagic, with negative values in the log of odds ratio (Fig. 2B). On the other hand, *Chloroflexi* (4% of total mesopelagic abundance), *Planctomycetes* (5%), *Euryarchaeota* (6%), *Marinimicrobia* (10%) and especially *Thaumarchaeota* (22%) were considered the representative phyla of the mesopelagic, with higher abundances than in the epipelagic and consequently positive log of odds ratios (Fig. 2B). Other less abundant phyla were more highly represented in the mesopelagic (27 out of the remaining 30 phyla not considered as the 10 most abundant) than in the epipelagic (18 of 30), suggesting a higher diversity in the deeper layers at this high taxonomical rank.

The Shannon’s alpha diversity index (H) calculated on all phyla ranged between 4.33 and 6.18 (Fig. 2C). The Shannon index increases as both richness (estimate of number of species, *n* in Fig. 2C) and evenness (relative distribution of species, shown as Pilou’s *J* in Fig. 2C) increase and shows that the mesopelagic samples had both significantly higher richness and evenness compared to the epipelagic samples, and especially when compared to the surface samples (ANOVA p < 0.01 n = 67, see posthoc results in Fig. 2C). In particular, the number of species observed in the DSL (1325 ± 48) remarkably doubled the species present in surface waters (590 ± 37).

Not surprisingly, the distribution of the samples in a Principal Coordinates Analysis (PCoA) of Bray–Curtis distances showed a clear segregation according to depth (Fig. 2D), with a strong separation between the samples from the surface and the DCM and a tight cluster of all the mesopelagic samples. This pattern was further supported by the distribution of biological and environmental variables over the PCoA ordination. Prokaryotic abundance, temperature and DOC were higher in the epipelagic layers, especially towards the surface. Salinity, inorganic nutrient concentrations (NO_3_^2−^, SiO_3_^2−^ and PO_4_^3−^), and prokaryotic cell size had higher values in the mesopelagic. Detailed vertical profiles of the abiotic and biotic variables are shown in Additional file 1: Fig. S3.

### DSL seasonal changes

**Fig. 3:**
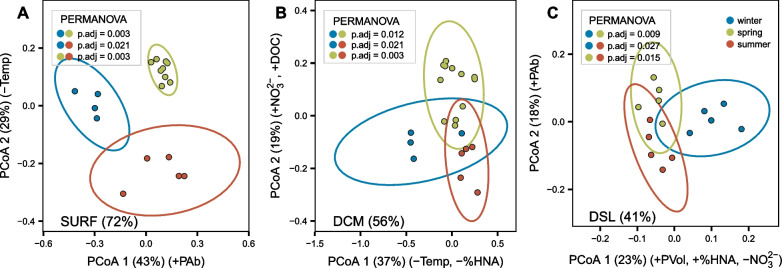
Sample distribution according to Principal Coordinates Analysis constructed on a Bray–Curtis dissimilarity matrix for SURF, DCM and DSL layers. The PERMANOVA test detected seasonal differences (p < 0.05) in all 3 layers considered and the pairwise comparisons between seasons are shown with adjusted p-values. On the bottom left, the percentage of variance explained by the first two axes is shown. Significant correlations between the axes and biotic or abiotic variables are shown **A** Surface (PCoA1 vs PAb r =  + 0.71; PCoA2 vs Temp r = −0.71), **B** Deep Chlorophyll Maximum (PCoA1 vs Temp r = −0.59; PCoA1 vs %HNA r = −0.53; PCoA2 vs NO_3_^2−^ r =  + 0.76; PCoA2 vs DOC r =  + 0.55) and **C** Deep scattering layer (PCoA1 vs PVol r =  + 0.71; PCoA1 vs %HNA r =  + 0.65; PCoA1 vs NO_3_^2−^ r = −0.93; PCoA2 vs PAb r = −0.69)

The seasonal analysis within the different layers was performed only for SURF, DCM and DSL, due to the absence of samples for the MS and MD layers in winter and summer. Within each layer, the samples clustered together according to the different sampling seasons in PCoA ordinations of Bray–Curtis distances (Fig. 3). These clusters were significant (p < 0.05) according to the pairwise PERMANOVA analysis in all three layers, but the differences between seasons were reduced with depth. The variance explained by the two most relevant axes decreased from representing 72% in the surface samples (Fig. 3A), to 56% in the DCM (Fig. 3B) and 41% in the DSL (Fig. 3C, in this case the third axis represented 10%). The main axes were correlated to different variables at each layer and can help explain the sample distribution. At the surface, the main drivers were prokaryotic abundance and temperature. At the DCM, the main drivers were temperature, %HNA and the concentrations of nitrate and DOC. At the DSL, nitrate had the strongest effect, but also prokaryotic size, prokaryotic abundance and %HNA (see correlation details in Fig. 3 caption). The PCoA was performed on the ASV matrix, but we could also detect the seasonal changes at the DSL for the annotated taxonomy at levels as high as phylum (PERMANOVA p < 0.05). In this case only the combination of winter vs summer was significantly different (padj = 0.027). We looked at the fluorescence-based biological index of DOM and found that the BIX was significantly higher in winter than in the other seasons at the DCM and DSL (one-way ANOVA), suggesting that more labile compounds with an autotrophic origin were available during that season.

There were clear seasonal differences in the behavior of environmental variables in the upper ocean (mainly temperature, salinity and oxygen), as shown in the profiles of Additional file 1: Fig. S3, as would be expected for the epipelagic. However, we also observed seasonal variability in the mesopelagic but no oceanographic phenomena were obvious during the cruises that could explain the differences in the mesopelagic variables (e.g. no intrusions of the Gulf of Aden Intermediate Waters with higher nutrient load).

Further focusing on the DSL, we evaluated the similarities and differences in diversity across the different seasons. The composition of the DSL was fairly homogeneous throughout all the collected samples at this depth. Shared ASVs across seasons identified with 3-way Venn diagrams represented 45% of total ASVs present in the DSL but accounted for over 90% of the relative sequence abundance (Additional file 1: Fig. S6). The most abundant group was the ammonia oxidizing archaea of the order *Nitrosopumilales* (phyla *Thaumarchaeota*) with a relative abundance of 22%. The next most prevalent group was the SAR11 clade (class *Alphaproteobacteria*, 11%), followed by unclassified orders of phylum *Marinimicrobia* (10%), SAR324 (class *Deltaproteobacteria*, 7%), UBA10353 (class *Gammaproteobacteria*, 5%), Marine Group II (class *Thermoplasmata*, 4%), *Rhodospirillales* (class *Alphaproteobacteria*, 3%), SAR202 (class *Dehalococcoidia*, 3%) and SAR86 (class *Gammaproteobacteria*, 2%).

**Fig. 4:**
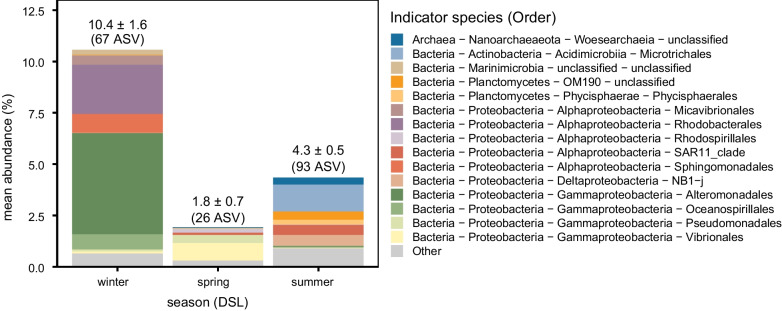
Mean sequence relative abundance of indicator species identified at the DSL for each season, color-coded by order. Only the top 15 orders are shown. The numbers over each bar indicate the mean ± SE sequence abundance and the number of indicator species (ASV) identified for each season

We analyzed each season’s indicator species at the DSL using the *indval* index [53]. This index considers the presence of a given ASV across samples and its relative abundance to determine if an ASV is strongly associated with a given group. ﻿The index is maximum (1) when the individuals of a given species are observed in all sites of only one group. We considered that an ASV was an indicator of season when p < 0.05 and *indval* > 0.5. We thus obtained 67 indicator ASVs for winter, 26 ASVs for spring and 93 ASVs for summer. Figure 4 shows the relative abundance of these indicator species at the order level for each of the seasons in the DSL. In winter, the indicator species represented slightly over 10% of the total abundance (10.4 ± 1.6%) and were dominated by *Alteromonadales* (class *Gammaproteobacteria*) and *Rhodobacterales* (class *Alphaproteobacteria*). In spring, however, indicator ASVs represented less than 2% of the total abundance (1.8 ± 0.7%) and the main contributor was *Vibrionales* (class *Gammaproteobacteria*). In summer, the indicator species yielded roughly 4% (4.3 ± 0.5%) and were represented by several clades including *Microtrichales* (class *Actinobacteria*), SAR11 (class *Alphaproteobacteria*) and clade NB1-j (class *Deltaproteobacteria*).

### DSL prokaryotic diversity

**Fig. 5:**
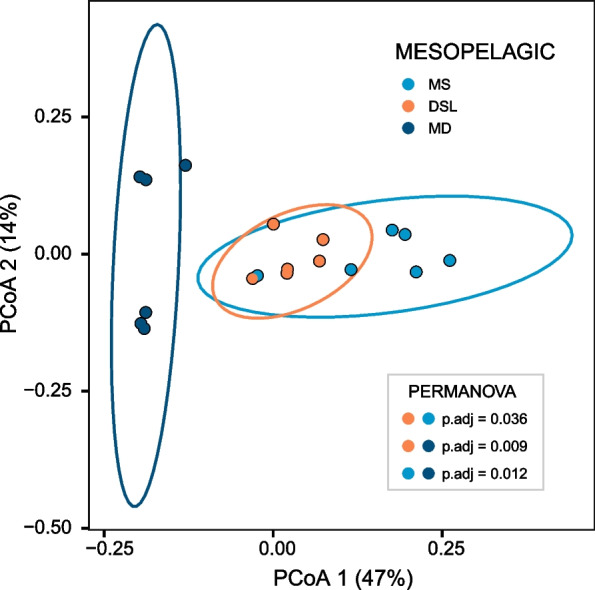
Sample distribution of mesopelagic samples in Principal Coordinate Analysis constructed on a Bray–Curtis dissimilarity matrix. The PERMANOVA test indicated an overall significant effect of the variable *layer* (p < 0.05), and the pairwise comparisons show differences between the three mesopelagic layers (MS, DSL, MD), see adjusted p-values in the figure

For spring, we further analyzed the diversity of the DSL by simultaneously comparing the DSL to the two layers located immediately above (MS) and below (MD) it. In Fig. 5 we show the PCoA distribution of the ASVs of spring samples for the mesopelagic layers (MS, DSL, MD) according to Bray–Curtis distances. The pairwise PERMANOVA comparisons indicated that all three layers were significantly different from each other, but especially the DSL and MS from MD (see p-value details in Fig. 5). It is important to note the difference in resolution between the data shown in Fig. 5, where samples are analyzed at the ASV sequence level, and the profiles shown in Fig. 2A, where the samples are analyzed after aggregating the sequences at high taxonomic levels (phyla and order). The direct sequence analysis of ASVs is more sensitive to differences between the communities of the mesopelagic layers. For example, the PERMANOVA analysis performed on the aggregated sequences at phylum level also identified differences between layers (DSL-MD and MS-MD) but it was not able to detect differences between DSL and MS.

**Fig. 6:**
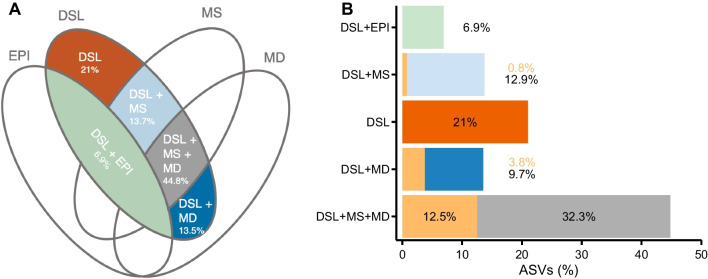
Sources of diversity at the DSL in spring. **A** 4-way Venn diagram schematic indicating the different diversity sources considered **B** Percentage of species (ASVs) associated with each Venn diagram intersection: in green are ASVs associated with sinking (EPI + DSL), in light blue ASVs shared between the shallow mesopelagic and DSL (MS + DSL), in dark orange ASVs that are unique to the DSL, in dark blue ASVs shared between the deep mesopelagic and the DSL (MD + DSL), in gray ASVs shared among the three mesopelagic depths (MS + DSL + MD) and in light orange are ASVs that are enriched in the DSL. The percentages shown here represent the mean values of the 4-way Venn diagrams run separately for each of the 5 spring depth profiles

For the next analysis we merged SURF and DCM samples and treated them as epipelagic samples (EPI). By using a 4-way Venn diagram (crossing the layers EPI, DSL, MD and MS) considering each spring depth profile as an independent sample, we were able to identify the sources of diversity at the DSL and their relative contribution in number of ASVs (Fig. 6). We have divided the main sources into: (1) epipelgaic ASVs (the ASVs at the DSL that were shared with SURF or DCM, and presumably have an epipelagic origin), (2) unique ASVs (the ASVs at the DSL that were not shared with any of the other layers) and (3) common mesopelagic ASVs (the ASVs at the DSL that are neither from the epipelagic nor unique). We further divided the mesopelagic partitioning into ASVs shared only with MS, ASVs shared only with MD and ASVs shared among the three mesopelagic layers (MS, MD, DSL). For the mesopelagic, we were able to identify ASVs that were not unique at the DSL but showed higher relative abundances in the DSL than in the adjacent layers (MS and MD), according to the results of ANOVA tests and Tukey post hoc differences in means. These ASVs are considered here as sequences that possibly have their niche in the DSL and have diffused toward the neighboring layers since there is no physical boundary separating the shallow and deep mesopelagic from the DSL.

Of the total number of ASVs identified at the DSL (n = 1190 ± 119), the ASVs with a mesopelagic origin (shared with any of the other mesopelagic layers: DSL + MS, DSL + MD or DSL + MD + MS in Fig. 6) dominated the diversity contribution at the DSL (72%). The diversity related to epipelagic ASVs accounted for 7% of ASVs (DSL + EPI in Fig. 6). The diversity identified as unique from the DSL was 21%. In addition, a 17% of the ASVs shared with either of the other mesopelagic layers had higher abundances in the DSL (sum of the light orange fractions in Fig. 6). Considering these sequences, the diversity that could be attributed to the DSL increased to 38%. The taxonomic composition at each of the five partitions can be consulted in Additional file 1: Fig. S7.

In order to identify the overall contribution of the DSL to the whole Red Sea mesopelagic diversity, we pooled all depth profiles together to look at all the ASVs that are unique to the DSL layer independently of the depth profile they are coming from. We identified that 21% of ASVs were unique to the DSL and represented 12% of the relative abundance of the mesopelagic. If the ASVs with higher abundances were included, we found that the DSL contributed to the mesopelagic diversity with 30% of species representing 32% of its relative abundance.

## Discussion

This is the first study to extensively analyze the mesopelagic microbial diversity along the longitudinal axis of the Red Sea. Indeed, most studies targeting prokaryotic diversity in the Red Sea have focused on the shallow epipelagic (surface and DCM) [54–56] or the deep brine pools [57–59], with only two including the mesopelagic or bathypelagic layers [60, 61] and only one mentioning specifically the DSL [1]. The DVM and the presence of DSLs are widespread across oceanic basins [4, 5] but their effect on prokaryotes remains poorly studied [27]. Here we focus on the 2 deepest echosounder layers in the Red Sea, composed mainly of the species *Vinciguerria* sp. and *Benthosema pterotum* [11]. We show that there is seasonal variability in the prokaryotic diversity of the DSL as well as differences with the other mesopelagic layers that surround it. We also identify the different sources contributing to the DSL diversity and conclude that around 21% was unique to the DSL (Fig. 6B).

Overall, the Red Sea diversity patterns showed changes from the epipelagic to the mesopelagic layers, with differing dominant groups and higher number of species in the latter, reaching a maximum around the depth of the DSL (Fig. 2C). The Shannon diversity index ranges observed (4–6) are similar to those obtained for the open ocean of the Global Ocean Sampling initiative [62] and for depth profiles of the northern Gulf of Mexico [63]. Similarly to Frank et al. [64] for samples in the North Atlantic and Walsh et al. [65] in the Equatorial and North Pacific, both richness and evenness increased in the mesopelagic. We should be cautious when comparing our estimates of alpha diversity to other diversity analyses since different techniques frequently yield different results, especially when comparing OTUs to ASVs [66]. Nonetheless, the general pattern indicates an increase in the diversity of the mesopelagic in different oceanic basins [64, 65, 67] as also shown for the Red Sea here.

The dominating groups in the mesopelagic where similar to those reported for the dark ocean (200 to 4000 m) [2, 27, 61, 64, 68–70] and included *Planctomycetes* (order *Phycispaherales*), *Chloroflexi* (clade SAR202), *Marinimicrobia*, *Gammaproteobacteria* (order UBA10353, *Alteromonadales*, SAR86, *Oceanospiralles*) *Alphaproteobacteria* (order SAR11, *Rhodospirillales*), *Deltaproteobacteria* (order SAR324) and *Thaumarchaeota* (order *Nitrosopumilales*). Some of these groups (especially heterotrophs) are not exclusive of the dark ocean (e.g. SAR11, *Alteromonadales*, *Rhodospirillales*), but others thrive in the mesopelagic conditions and are much more abundant than in upper layers (e.g. SAR324, SAR202, *Nitrosopumilales*) [71]. It may not be surprising that diversity increases in the deeper waters, since the absence of light, increase of inorganic nutrients and low oxygen levels, fuel a multitude of different strategies to obtain energy and carbon [72, 73], apart from the most extended heterotrophic lifestyle. For example, some members of the *Planctomycetes* are responsible for anammox processes (in near to anoxic waters) while others have developed strategies to colonize marine snow [72, 74, 75]. *Chloroflexi* members show diverse metabolisms including anoxygenic phototrophy, but deep members such as SAR202 are aerobic thermophiles and heterotrophs that metabolize organosulfur compounds [74, 76] and could participate in the metabolism of recalcitrant DOM [77, 78]. *Marinimicrobia* (formerly known as SAR406) is yet another highly diverse phylum that also peaks in the mesopelagic [74] and low oxygen areas [79], where several potential pathways using nitrogen and sulfur may contribute to link their metabolism to other groups such as *Planctomycetes* and *Thaumarchaeota* [79]. Many of the deep groups have also potential to incorporate dissolved inorganic carbon, including the *Deltaproteobacteria* SAR324, *Planctomycetes*, and also members of *Gammaproteobacteria* (*Oceanospirillaes*, *Alteromonadales*) and even SAR11 [71, 80, 81], further illustrating the complexity of the processes taking place at the deep ocean.

All the aforementioned groups were present in the Red Sea, but their abundances were not comparable to the order *Nitrosopumilales* (*Thaumarchaeota*, formerly part of *Crenarchaeota* and also known as Marine Group I Archaea, [82]), which represented on its own 22% of all the microbial abundance of the mesopelagic and 74% of all Archaeal sequences (99% of *Thaumarchaeota*). Archaea have been seen to increase considerably in the aphotic ocean, with observations of the *Crenarchaeota*/MG-I/*Thaumarchaeota* phylum reaching up to 40% of prokaryotic abundance [83] but more often estimated around 20% [68, 84]. Most members of *Thaumarchaeota* are ammonia oxidizers [72, 85] and have shown potential to incorporate inorganic carbon [74, 86] and exude organic compounds [87], but have also shown heterotrophy and mixotrophy strategies [88]. Their high abundance in the deep ocean may indicate substantial contributions to the dark microbial metabolism, particularly in the cycling of nitrogen, carbon and even phosphate [89]. A previous study at the Red Sea reported even higher concentrations of *Thaumarchaeota* in deep waters (40–50% of the order *Nitrosopumilales*) [1] but these numbers seem overestimated, probably because the primer pair used (515F/909R, [90]) was not specifically tested and developed to detect pelagic marine bacteria and archaea, with special emphasis on *Thaumarchaeota*, as the primer used here (515-YF/926R [31]).

The mesopelagic realm of the Red Sea differs from other marine basins in its relatively low residence time of deep waters (36–90 years) [18] and its unparalleled high average temperature (Additional file 1: Fig. S2). It is largely composed of a very homogeneous water mass from 200–250 m to the bottom known as the Red Sea Deep Water mass (RSDW, Additional file 1: Fig. S2) [18, 50]. We observed that some physico-chemical properties were variable (DOC, oxygen and especially nitrate and phosphate concentrations, which showed lower values in winter), suggesting a less uniform layer than generally assumed for the mesopelagic. Several well studied atmospheric and oceanographic phenomena (Gulf of Aden advection, mesoscale eddies, seasonal stratification shoaling, monsoon reverse winds) are responsible for the variability in Red Sea shallow water masses [91–93] but much less is known about the deep circulation [18] and especially about the nutrient dynamics in deep layers, as to explain the lower nutrient values observed in winter. Since no intrusions of water masses different from the RSDW were evident from the analysis of the CTD profiles and TS diagrams (Additional file 1: Fig. S2), we could assume that the whole mesopelagic water mass analyzed was fairly homogeneous in its thermohaline properties. In much larger surveys, different deep prokaryotic communities have been associated with distinct water masses [64] and oceanographic basins [68]. Consequently, the overall uniform taxonomic composition of the Red Sea mesopelagic aligns with the notion of a homogeneous water mass, such as the RSDW (Additional file 1: Fig. S3).

The greatest limitation to prokaryotic activity in the mesopelagic has been attributed to the low concentration of DOC [2, 24] and/or to its refractory nature [94, 95]. The average mesopelagic DOC concentrations in our cruises (54.4 ± 1.2 μmol L^−1^) matched the values observed in the central Red Sea in a previous study (50.7 ± 4.1 μmol L^−1^) [26]. DOC in the Red Sea mesopelagic are in the lower range of the values found in other mesopelagic basins (50–80 μmol L^−1^ [96, 97]) but were well above the minimum concentration that can hypothetically support heterotrophic prokaryotic metabolism (30.7 ± 5.4 μmol L^−1^ [24]). Indeed, DOC from the mesopelagic zone of the central Red Sea is incorporated into heterotrophic bacterial biomass with even higher rates than in the surface waters [1]. In general, the organic carbon present in the deep ocean derives from three main sources: passive flux (sinking of particles), active flux (migrating organisms) and mixing [2]. However, the export of carbon to deeper waters does not seem to match the carbon demand of deep prokaryotes, meaning that some sources remain unaccounted for or are underestimated [98, 99]. For example, as mentioned above, ammonia oxidizers (like the abundant *Thaumarchaeota*) or *Planctomycetes*, have the ability of dark inorganic carbon fixation [81, 100] and may contribute to the organic carbon pool of the deep ocean at rates similar to the heterotrophic consumption [101]. On the other hand, vertically migrating organisms are a potential source of labile DOC and ammonia [102, 103] that can be released at the depths where they concentrate during the day (~ 490–600 m). These compounds can be quickly consumed by specific microbes [104–106] that help shape the prokaryotic community structure at the DSL. The amount of the organic matter exported by DVM remains poorly quantified [20] but we show that its effects may be observed through the microbial community at the DSL.

In this study, we were able to detect seasonal differences in the microbial community composition at the DSL (Fig. 3) and identify indicator prokaryotes for each of the 3 seasons assessed (Fig. 4). According to our results, winter samples seemed to differ most from spring and summer samples. They also had the highest contribution of indicator ASVs to total number of reads (10%). However, in terms of number of species, summer had the greatest number of indicator ASVs (87). These seasonal patterns may be potentially related to changes in the seasonal composition and quantity of the available dissolved organic matter at depth as well as to the contribution of differential sinking particles. Both could change in response to seasonal changes in the food sources for migrating fish, from phytoplankton [107] to zooplankton [108]. This is supported by the fact that the presence of labile DOM (as indicated by the BIX index) changed between seasons at the DSL. Surprisingly, variations of over 15 µmol l^−1^ in DOC concentrations have been observed in the mesopelagic of the central Red Sea and have been related to seasonal differences in the downward export of DOC from the epipelagic through passive physical mechanisms (concentration gradients) as well as to the active role of diel migrating organisms [26].

We were also able to detect differences in the microbial composition when comparing the DSL with the mesopelagic depths located immediately above and below the layer occupied by the fish (450–600 m) (See Additional file 1: Fig. S1 for reference of the sampling depths collected during spring, outside of the DSL). The deep mesopelagic (MD) (between 750–900 m) showed a different community structure and was apparently more isolated from the DSL and the shallow mesopelagic MS (250–300 m), although the 3 layers shared virtually the same physico-chemical properties within a given season, and hence, the same water mass (Additional file 1: Fig. S2). We hypothesize that the greater similarity between the shallow mesopelagic and the DSL can be attributed to the diel migration of fish, since the fish need to cross the MS layer twice every day to reach the surface and return to the DSL, potentially releasing, though for small periods, the same organic substances as at the DSL. In agreement, [1] also found that heterotrophic prokaryotes at an intermediate water layer between the surface and the DSL of the Red Sea showed similar growth rates to those at the DSL.

Finally, we were able to quantify the diversity introduced to the mesopelagic by the DSL. The general conditions of the mesopelagic zone (almost complete darkness, low oxygen concentration, low organic inputs, high hydrostatic pressure, and high inorganic nutrient concentrations) make the overall prokaryotic diversity of the whole layer very consistent (Fig. 2). We could not discriminate between different environmental variables explaining unequivocally the prokaryotic community structure of the DSL but we could break down the origin of the sequences at the DSL. On average for the 5 depth profiles considered, 72% of the species present in the DSL are shared across the mesopelagic, compared to the 21% that can be directly attributed to the DSL because they are found here uniquely. This one-fifth of exclusive ASVs is in itself striking, but this percentage is almost doubled (38%) if we add the ASVs that are observed more frequently in the DSL than in either of the two other mesopelagic depths. Considering all the mesopelagic samples together, the diversity likely introduced by the DSL community to the mesopelagic increases and would represent 30% of ASVs and 32% of the abundance. The 2 DSLs have a width of 150–200 m in the central Red Sea [9], and therefore, less than 20% of the total volume of the mesopelagic would harbor almost one-third of the biodiversity of bacteria and archaea, suggesting an overlooked hotspot for microbial diversity in the twilight zone.

The sequencing of 16S rRNA gene allows for a general overview of the microbial community composition at a given site. It represents a snapshot but does not have enough resolution for the observation of the fast metabolic changes (in time) that might occur in the presence of the fishes. Therefore, despite the relatively high amount of ASVs shared with the rest of the mesopelagic, an enhancement of the metabolism of prokaryotes in response to the organic matter pulses supplied during the day at the DSL would be expected. Indeed, the prokaryotes at the DSL have shown higher growth efficiencies than surface prokaryotes during the day [1], indicating probable higher metabolic rates due to the increased organic sources. In a different study, García et al. [19] showed clear and parallel diel cycles in DOC and prokaryotes cell abundance and physiological structure in both the epipelagic and mesopelagic. Particularly at the depths between 400 and 500 m, an increase of high molecular weight dissolved organic compounds was observed during the day and correlated to an increase in heterotrophic prokaryotic abundance. Recently, [109] have demonstrated significantly larger responses of heterotrophic prokaryotes growth in the midday incubation relative to the incubation conducted at night with predator-free seawater collected from the DSL. These studies and our own, should provide evidence that the DVM of fish promotes activity as well as diversity at the DSL. However, more metabolic based approaches (production and respiration rates, nitrification rates, transcriptomics) as well as export measurements and isotopic DOC fingerprinting, are suggested for future research to assess the role of the microbial community in the DSL and quantify their effects on the global carbon and nutrient cycles. In order to gain a better understanding of the mesopelagic and its variability in general, we recommend adopting higher-resolution sampling strategies within the mesopelagic (e.g. at least distinguishing between the layers above and below the DSL).

In summary, this is the first extensive report focused on the prokaryotic plankton diversity of the deep scattering layer in the Red Sea, and one of the pioneering studies globally, after a recent study in the South China Sea [27]. We have shown that the overall mesopelagic zone has a fairly homogeneous prokaryotic composition at high taxonomic levels, but important differences arose at the individual species (ASVs) resolution, both when comparing different depths within the mesopelagic and (three) different seasons. The demonstration of a direct correlation between the diel migration of large organisms and prokaryotic diversity at the DSL requires of more metabolic based approaches. However, we conclude that, on average, 21% of the ASV diversity found at the DSL is exclusive to that layer and that overall (considering all depth profiles together and also ASVs with higher abundance in the DSL), the DSL contributes almost one third of the diversity of the whole mesopelagic layer in the Red Sea.

### Supplementary Information

**Additional file 1. Fig. S1** shows the echosounder profiles collected during samplings; **Fig. S2** shows the TS diagram of all depth profiles; **Fig. S3** shows the depth profiles of environmental and biological variables collected during the study; **Fig. S4** shows the individual 16S diversity profiles of each sample at Phylum level; **Fig. S5** shows the mean sequence abundances of all samples; **Fig. S6** shows the seasonal distribution of diversity at the DSL; **Fig. S7** shows the contribution of sequences to the DSL by source layer.

## Data Availability

The raw sequences used in this study are available at https://www.ebi.ac.uk/ena/browser/view/PRJEB49545 as 67 paired fastq sequences with consecutive accession numbers: ERX7411972–ERX7412038. The metadata, processed sequence counts and taxonomic affiliation data that support the findings of this study are openly available in ZENODO at https://doi.org/10.5281/zenodo.5816123 (Huete-Stauffer et al. 2022). The code to replicate the main figures is available in the following repository https://github.com/tamaramegan/RedSea_DSL.

## Abbreviations

ASV: Amplicon sequence variant
DCM: Deep chlorophyll maximum
DOC: Dissolved organic carbon
DOM: Dissolved organic matter
DSL: Deep scattering layer
DVM: Diel vertical migration
EPI: Epipelagic
BIX: Biologic Index of DOM
MS: Shallow mesopelagic
MD: Deep mesopelagic
POC: Particulate organic carbon
POM: Particulate organic matter
