# Supplementary material for: Increased prokaryotic diversity in the Red Sea deep scattering layer
Source: Environ Microbiome. 2023 Dec 14;18:87. doi: 10.1186/s40793-023-00542-5 (PMC10722844; doi:10.1186/s40793-023-00542-5)
Supplement: Supplementary file 1 — Additional file 1. Fig. S1 shows the echosounder profiles collected during samplings; Fig. S2 shows the TS diagram of all depth profiles; Fig. S3 shows the depth profiles of environmental and biological variables collected during the study; Fig. S4 shows the individual 16S diversity profiles of each sample at Phylum level; Fig. S5 shows the mean sequence abundances of all samples; Fig. S6 shows the seasonal distribution of diversity at the DSL; Fig. S7 shows the contribution of sequences to the DSL by source layer. [file 40793_2023_542_MOESM1_ESM.pdf]

Supplementary material

Increased prokaryotic diversity in the Red Sea deep scattering layer

Tamara Megan Huete-Stauffer, Logares R, Ansari MI, Rostad A, Calleja M Ll, Morán XAG

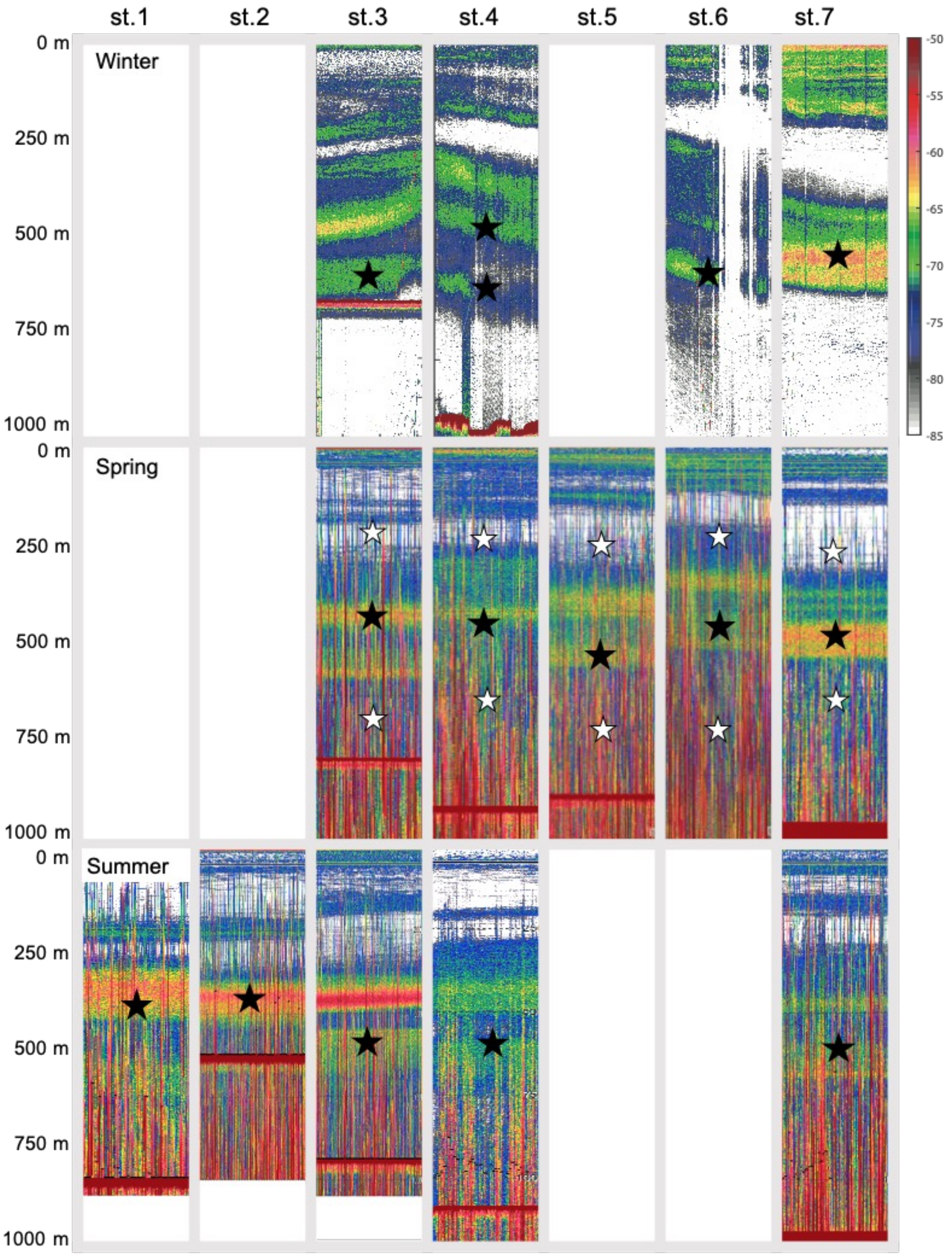

Fig. S1. Echosounder profiles (38kHZ) at each station at the time of sampling. Winter profiles were made with a hull-mounted Simrad EK60 while Spring and Summer were made with a portable Simrad WBAT. Black stars indicate the depth of sample collection at the deep scattering layer and white stars indicate the layers MS and MD, above and below the DSL.

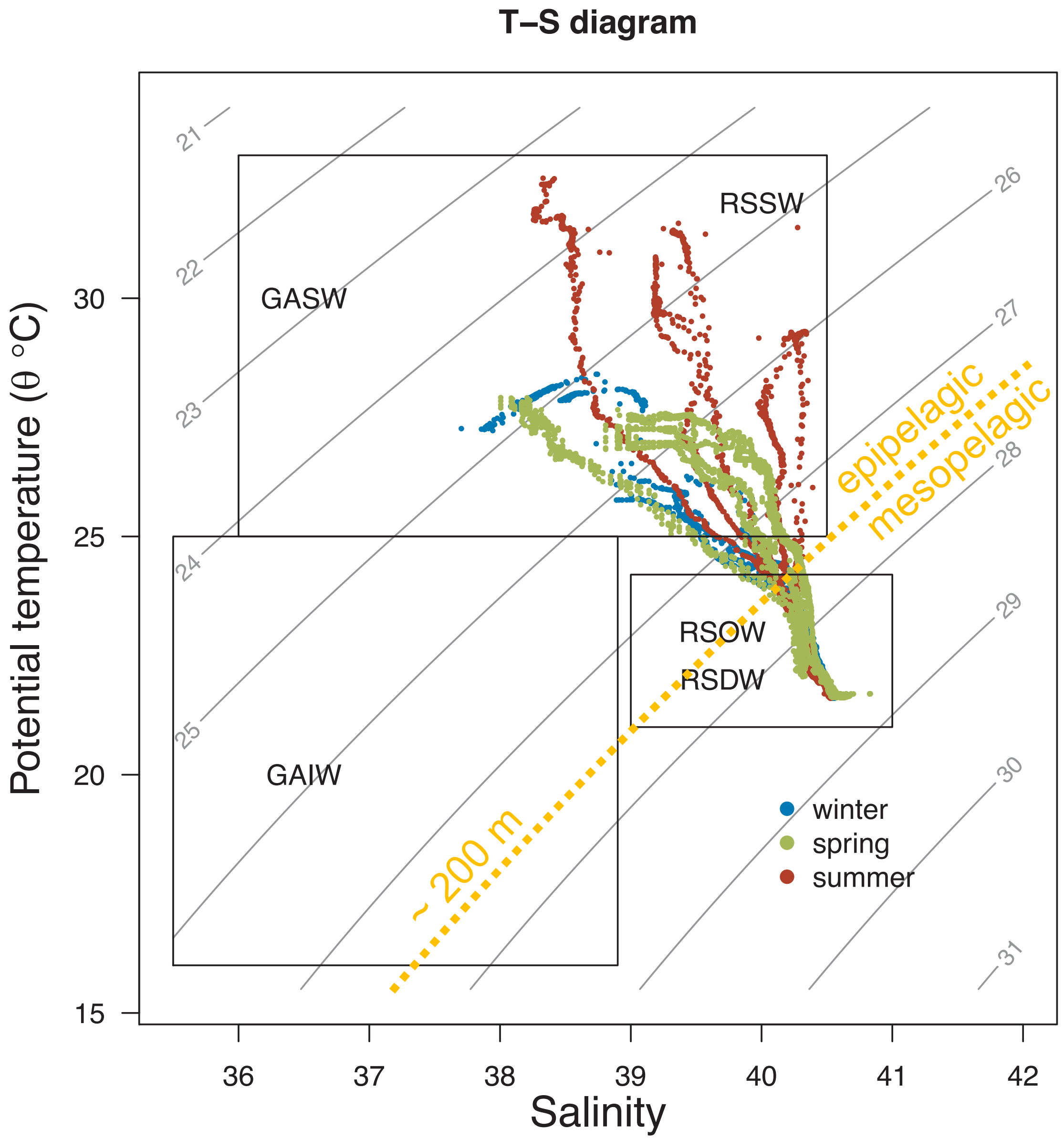

Fig. S2. Temperature-Salinity (T-S) diagram of all profiles collected in the study. Boxes enclose the expected T-S values for different Red Sea water masses. RSSW: Red Sea Surface Water; RSOW: Red Sea Outflow Water; RSDW: Red Sea Deep Water; GASW: Gulf of Aden Surface Water; GAIW: Gulf of Aden Intermediate Water. The dotted yellow line highlights the isopycnal 27.5 Kg m^{-3} that represents the cut between the epipelagic and mesopelagic and is found around 200 m. During the sampling period, seasonal changes and variability was observed in epipelagic waters (above isopycnal ~ 27.5 Kg m^{-3}), while mesopelagic waters remained constant in their thermohaline properties. We did not observe intrusions from the Gulf of Aden (GASW or GAIW).

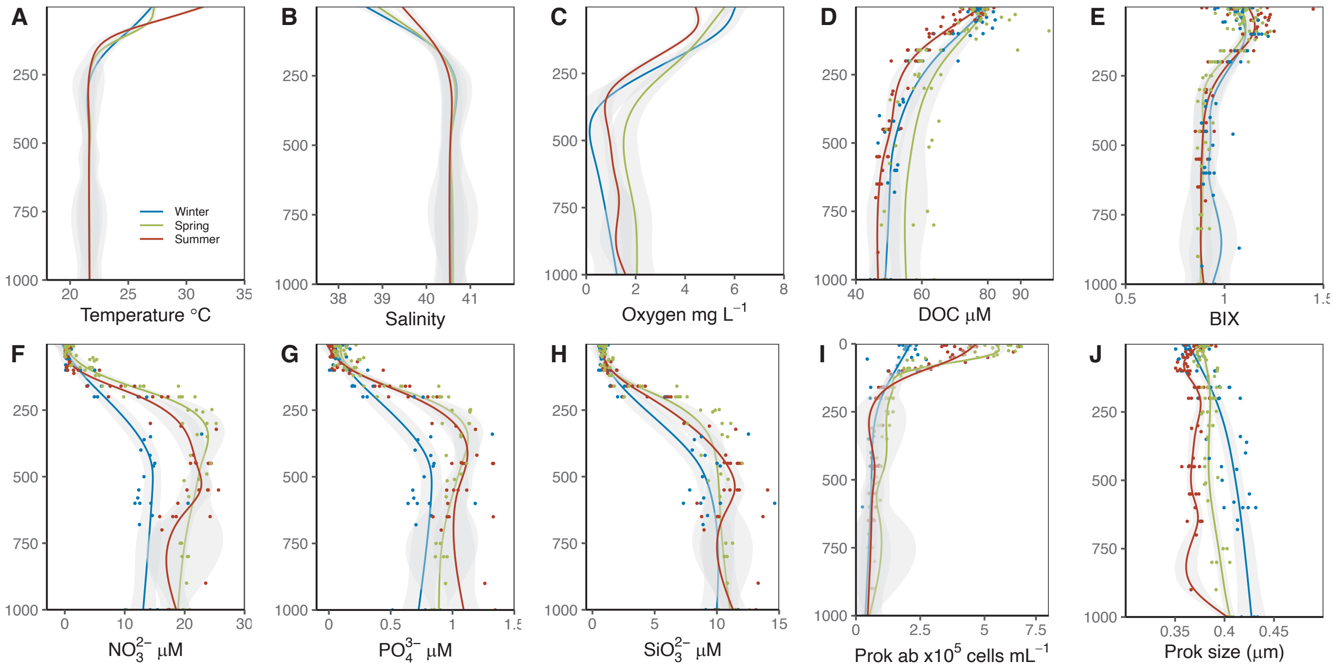

Fig. S3. Depth profiles of environmental and biological variables collected during the study. The data for each variable was fitted to a generalized additive model (GAM) with differing intercepts for each season. Dots represent the discrete data points. Shadowed areas in grey indicate 95% confidence intervals. Panels A (Temperature), B (Salinity) and C (Oxygen) represent continuous CTD data.

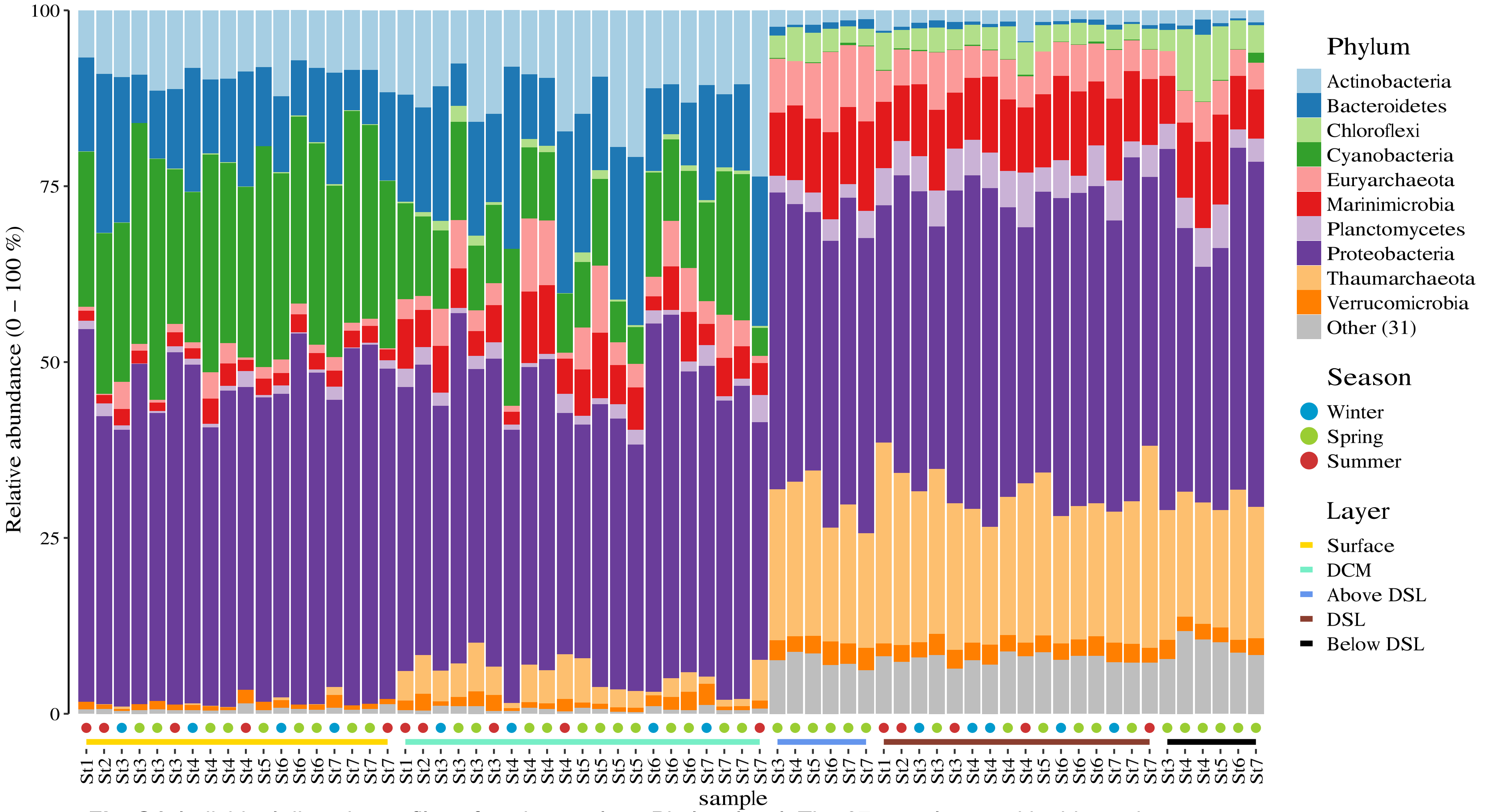

Fig. S4. Individual diversity profiles of each sample at Phylum level. The 67 samples used in this study are shown aggregated by layer and matched to the station where they were collected and thduring which season.

	Phylum	mean sequence abundance (%)
1	Proteobacteria (Alpha)	24.8
2	Proteobacteria (Gamma)	12.7
3	Cyanobacteria	10.9
4	Thaumarchaeota	9.7
5	Bacteriodetes	8.4
6	Actinobacteria	7.4
7	Marinimicrobia (SAR406)	6.7
8	Proteobacteria (Delta)	5.3
9	Euryarchaeota	4.3
10	Planctomycetes	2.6
11	Chloroflexi	1.9
12	Verrucomicrobia	1.7
13	Acidobacteria	0.78
14	Nitrospirae	0.60
15	Gemmatimonadetes	0.60
16	Nanoarchaeaeota	0.44
17	Nitrospinae	0.42
18	Dadabacteria	0.34
19	PAUC34f	0.25
20	Kiritimatiellaeota	0.08
21	Margulisbacteria	0.05
22	Proteobacteria (NA)	0.04
23	Patescibacteria	0.04
24	Entotheonellaeota	0.04
25	Lentisphaerae	0.03
26	WPS-2	0.03
27	AncK6	0.03
28	Schekmanbacteria	0.02
29	Chlamydiae	0.01
30	Dependentiae	0.005
31	Spirochaetes	0.005
32	Tenericutes	0.004
33	Poribacteria	0.004
34	Deinococcus-Thermus	0.004
35	Firmicutes	0.002
36	Crenarchaeota	0.001
37	Armatimonadetes	0.0009
38	BRC1	0.0009
39	Omnitrophicaeota	0.0002
40	Epsilonbacteraeota	0.0002
41	Hydrogenedentes	0.0002
42	Fibrobacteres	0.0002
43	Fusobacteria	0.0001

Fig. S5. Phyla mean sequence abundance included in Fig. 2A and 2B. (note that Phylum Proteobacteria has been split into its Orders). Groups with average abundance above 1% have been included in Fig. 2

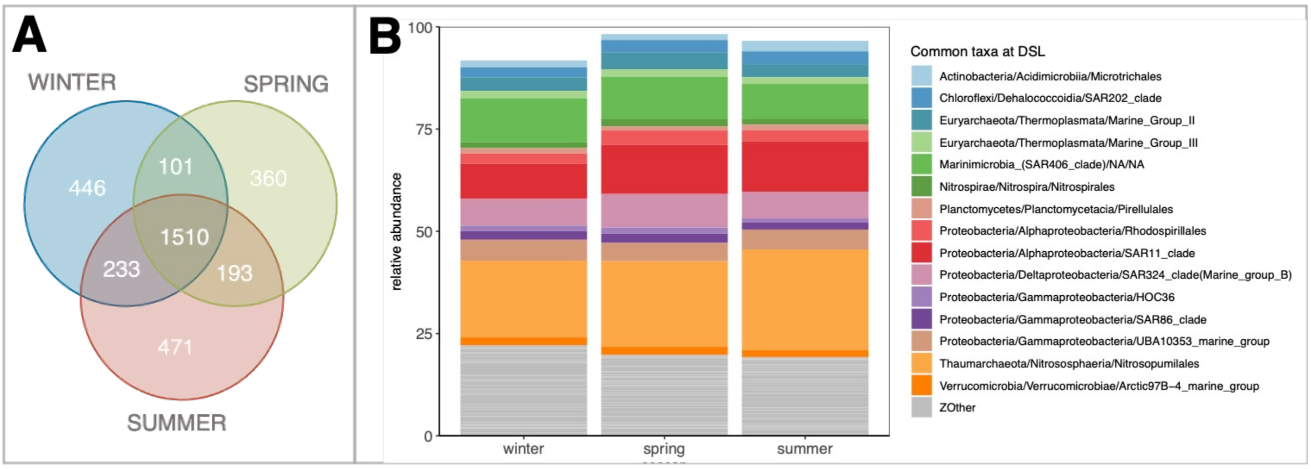

Fig. S6. Seasonal distribution of diversity at the DSL. **A:** 3-way Venn diagram showing the unique and shared ASVs for each season**. B:** Mean relative abundance of the shared sequences in each season annotated at Class level.

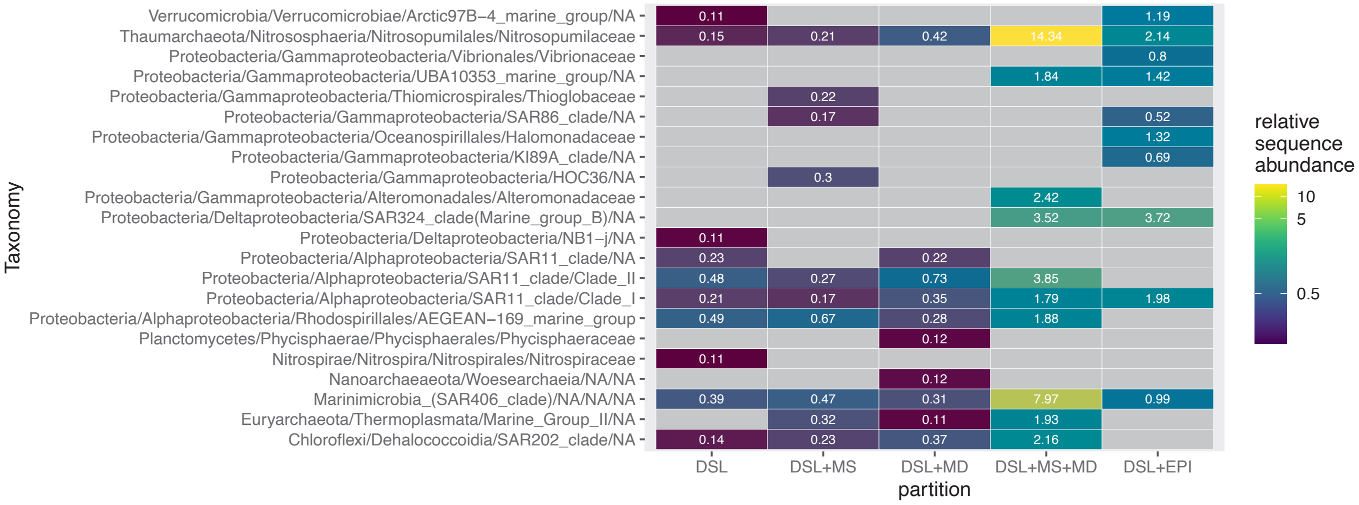

Fig. S7. Diversity distribution by source at the DSL. The heatmap shows the relative sequence abundance of the top 10 taxa annotated to Family from each of the 4-way Venn diagram partitions analyzed in Fig. 6.
